# Tetraspanins as Potential Modulators of Glutamatergic Synaptic Function

**DOI:** 10.3389/fnmol.2021.801882

**Published:** 2022-01-03

**Authors:** Amina Becic, Jennifer Leifeld, Javeria Shaukat, Michael Hollmann

**Affiliations:** Department of Biochemistry I – Receptor Biochemistry, Faculty of Chemistry and Biochemistry, Ruhr University Bochum, Bochum, Germany

**Keywords:** tetraspanins, CNS, synaptic function, glutamate receptor, modulator structures

## Abstract

Tetraspanins (Tspans) comprise a membrane protein family structurally defined by four transmembrane domains and intracellular N and C termini that is found in almost all cell types and tissues of eukaryotes. Moreover, they are involved in a bewildering multitude of diverse biological processes such as cell adhesion, motility, protein trafficking, signaling, proliferation, and regulation of the immune system. Beside their physiological roles, they are linked to many pathophysiological phenomena, including tumor progression regulation, HIV-1 replication, diabetes, and hepatitis. Tetraspanins are involved in the formation of extensive protein networks, through interactions not only with themselves but also with numerous other specific proteins, including regulatory proteins in the central nervous system (CNS). Interestingly, recent studies showed that Tspan7 impacts dendritic spine formation, glutamatergic synaptic transmission and plasticity, and that Tspan6 is correlated with epilepsy and intellectual disability (formerly known as mental retardation), highlighting the importance of particular tetraspanins and their involvement in critical processes in the CNS. In this review, we summarize the current knowledge of tetraspanin functions in the brain, with a particular focus on their impact on glutamatergic neurotransmission. In addition, we compare available resolved structures of tetraspanin family members to those of auxiliary proteins of glutamate receptors that are known for their modulatory effects.

## Introduction

A large family of abundantly expressed transmembrane proteins, found in all multicellular eukaryotes and comprising 33 members in humans, create an important protein network involved in a wide range of cellular processes such as cell proliferation, adhesion, signaling, fusion, and migration (Boucheix and Rubinstein, 2001). Back in 1988, a melanoma-associated antigen (ME491) was identified to consist of four transmembrane domains (TMs) (Hotta et al., 1988). Two years later its sequence homology to the Sm23 antigen found in the parasitic trematode *Schistosoma mansoni* was recognized (Wright et al., 1990). Further investigations revealed that the proteins CD37 (Classon et al., 1989) and TAPA-1 (target of the antiproliferative antibody 1, later denoted as CD81) (Oren et al., 1990) also share structural similarity. For classification purposes, the term “tetraspanins” was proposed for all members of the transmembrane four superfamily (TM4SF) (Maecker et al., 1997) (see Table 1 for overview of tetraspanin nomenclature). These proteins are believed to function through their shared exceptional ability to interact with each other and numerous partner proteins to create a dynamic network of interactions known as the “tetraspanin

web” or “tetraspanin-enriched microdomains” (TEMs) on the cell surface (Hemler, 2003; Charrin et al., 2009; Dornier et al., 2012). Consequently, tetraspanins are also regarded as molecular organizers (Boucheix and Rubinstein, 2001). For example, the interaction of tetraspanins with integrins has been well documented (Schmidt et al., 1996; Bassani and Cingolani, 2012). Integrins are cell adhesion molecules consisting of an α- and a β-subunit and can mediate both cell–cell and cell–extracellular matrix interactions (Hynes, 2002; Bassani and Cingolani, 2012). In this contect, tetraspanins, in the form of TEMs, are believed to exert a scaffolding function and organize proteins spatially and temporally in biological membranes (Perot and Ménager, 2020). For example, TEMs may be enriched in integrins and thus act as functional units involved in cell adhesion (Termini and Gillette, 2017; Perot and Ménager, 2020). With respect to the role of TEMs in the brain, interactions between Tspan28 (CD81) and Tspan29 (CD9) with integrins α3β1 and α6β1 have been shown to influence neurite growth (Schmidt et al., 1996; Stipp and Hemler, 2000; Bassani and Cingolani, 2012). Thus, these distinct units organized by tetraspanins play diverse roles in a variety of biological processes such as in viral infections, in cell–cell adhesion through interplay with the aforementioned integrins, and in cancer development and metastasis (Perot and Ménager, 2020). Viruses have been reported to preferentially enter cells via TEMs, which presumably can happen with or without direct binding to tetraspanins as receptors for virus entry (Pileri et al., 1998; Martin et al., 2005; Charrin et al., 2009; He et al., 2013; Hantak et al., 2019).

**TABLE 1:** Summary of human tetraspanins with their alternative names, tissue specificity and the functions.

Name	Alternative names *	Tissue specificity*	Protein function
Tetraspanin1	TSPAN-1, NET-1	Intestine, testis	Regulation of cell development, activation, growth and motility (Liu et al., 2019; Wang L. et al., 2021). Prognostic role in prostate (Stinnesbeck et al., 2021), pancreatic (Ma C. et al., 2021), and cervical (Wollscheid et al., 2002) cancer
Tetraspanin2	TSPAN-2, FLJ12082, TSN2	Smooth muscle	Contribution to oligodendrocyte differentiation into myelin-forming glia (Yaseen et al., 2017). Modulation of microglial cells (Reynolds and Mahajan, 2020). Association with migraine (Esserlind et al., 2013)
Tetraspanin3	TSPAN-3, TM4-A, TM4SF8	Low tissue specificity	Regulation of the expression of ADAM10, presenilin and the amyloid precursor protein (Seipold and Saftig, 2016). Associated with progression of acute myeloid leukemia (Kwon et al., 2015; Yang et al., 2016; Sun et al., 2020; Zhang Z. Y. et al., 2020)
Tetraspanin4	TSPAN-4, NAG-2, TM4SF7	Low tissue specificity	Potential biomarker in hepatocellular carcinoma and plays a critical role in promoting cancer cell proliferation (Li et al., 2021). Interacts with histamine H4 receptor (Ma X. et al., 2021)
Tetraspanin5	TSPAN-5, NET-4, TM4SF9	Brain and ovary	Promotes metastasis of hepatocellular carcinoma through Notch signaling (Xie et al., 2021). Involved in dendritic spine maturation process (Moretto et al., 2019). Plays a role in regulation of ADAM10 compartmentalization (Jouannet et al., 2016) and trafficking (Dornier et al., 2012) to the cell surface
Tetraspanin6	TSPAN-6, T245, TM4SF6	Salivary gland	Regulator of carcinogenesis in colorectal cancer (Andrijes et al., 2021), retinoic acid-inducible gene I-like receptor-mediated immune signaling (Wang et al., 2012) and Amyloid Precursor Protein metabolism (Guix et al., 2017)
Tetraspanin7	TSPAN-7, A15, CD231, DXS1692E, MRX58, MXS1, TALLA-1, TM4SF2	Brain	Involved in HIV-1 host-virus interaction (Ménager, 2017; Perot et al., 2020) regulates AMPA receptor trafficking (Bassani et al., 2012) and is autoantibody target in type 1 diabetes (McLaughlin et al., 2020)
Tetraspanin8	TSPAN-8, CO-029, TM4SF3	Intestine	Target candidate for immunotherapy of pancreatic adenocarcinoma (Schäfer et al., 2021). Highly expressed in renal carcinoma (Tang et al., 2020) and involved in lung adenocarcinoma migration (Xu et al., 2020)
Tetraspanin9	TSPAN9, NET-5	Brain, heart muscle	Regulates gastric cancer cell migration and invasion (Li et al., 2016; Qi et al., 2019) platelet function (Protty et al., 2009) and modulates the early endosome in virus entry (Stiles and Kielian, 2016)
Tetraspanin10	TSPAN10, OCSP	Retina	Involved in trafficking regulation of the transmembrane metalloprotease ADAM10 (Dornier et al., 2012). Genetic variant within the TSPAN10 gene is associated with strabismus (Plotnikov et al., 2019)
Tetraspanin11	TSPAN11	Intestine	Participates in determining the direction of bone matrix organization (Nakanishi et al., 2019)
Tetraspanin12	TSPAN12, NET-2, TM4SF12	Low tissue specificity	Involved in retinal vascularization by regulating norrin (NDP) signal transduction (Junge et al., 2009). Promotes ADAM10 maturation, facilitating ADAM10-dependent proteolysis of APP (Xu et al., 2009). Heterozygous mutation in TSPAN12 gene is associated with familial exudative vitreoretinopathy (Carroll and Kim, 2019)
Tetraspanin13	TSPAN13, NET-6, TM4SF13	Low tissue specificity	Potential marker indicating the outcome of breast cancer (Jiang et al., 2019). Involved in pathophysiology of thyroid cancer (Li et al., 2019)
Tetraspanin14	TSPAN14, DC-TM4F2, MGC11352, TM4SF14	Low tissue specificity	Regulates maturation and trafficking of the transmembrane metalloprotease ADAM10 (Dornier et al., 2012; Haining et al., 2017; Moretto et al., 2019; Noy et al., 2019)
Tetraspanin15	TSPAN15, NET-7, TM4SF15	Low tissue specificity	Essential subunit of an ADAM10 scissor complex (Koo et al., 2020). Overexpression positively regulates development oral squamous cell carcinoma (Hiroshima et al., 2019). Stemness-related marker in hepatocellular carcinoma (Sidahmed-Adrar et al., 2019)
Tetraspanin16	TSPAN16, TM-8, TM4-B, TM4SF16	Blood, testis	Under-expressed in acute lymphoblastic leukemia (Juric et al., 2007)
Tetraspanin17	TSPAN17, FBX23, FBXO23, TM4SF17	Low tissue specificity	Involved in regulation of ADAM10 trafficking (Dornier et al., 2012; Reyat et al., 2017). Decreased levels associated with improved survival in glioblastoma multiforme (Guo et al., 2019)
Tetraspanin18	TSPAN18	Low tissue specificity	Regulator of thrombo-inflamation (Noy et al., 2019; Gavin et al., 2020). Involved in the development of psychotic symptoms and schizophrenia (Yuan et al., 2013; Zhang et al., 2015; Liu et al., 2016; Wu et al., 2016)
Tetraspanin19	TSPAN19	Lung, pituitary gland	Specific function unclear
Tetraspanin20	TSPAN20, UPK1B, UPK1, Uroplakin 1B	Placenta, urinary bladder	Plays an important role in normal bladder epithelial physiology (Wu et al., 1995; Lobban et al., 1998). Involved in renal cell carcinoma (Yuasa et al., 1998; Finch et al., 1999; Wang et al., 2018; Zhang Z. Y. et al., 2020) and urinary tract inflammation prediction (Bulut et al., 2014)
Tetraspanin21	TSPAN21, UPK1A, Uroplakin 1A	Prostate, urinary bladder	Plays an important role in normal bladder epithelial physiology (Wu et al., 1995; Lobban et al., 1998; Ogawa et al., 1999; Hall et al., 2005). Upregulation connected with lung cancer cells (Byun et al., 2020), downregulation enhances apoptosis of bladder carcinoma cells (Zhu et al., 2015) and reduced expression associated with gastric adenocarcinoma (Zheng et al., 2014)
Tetraspanin22	TSPAN22, PRPH2, CACD2, rd2, RDS, RP7, Peripherin 2	Retina	Essential for retina photoreceptor outer segment disk morphogenesis (Donato et al., 2021) and involved in retinal degeneration (Travis et al., 1991), macular dystrophy (Kumaran et al., 1993; Çavdarli et al., 2020) and retinitis pigmentosa (Kajiwara et al., 1991; Farrar et al., 1992; Jordan et al., 1992; Li et al., 2021)
Tetraspanin23	TSPAN23, ROM1, ROM, Retinal outer segment membrane protein 1	Retina	Plays a role in rod outer segment (ROS) morphogenesis (Conley et al., 2017). Involved in retinitis pigmentosa (Kajiwara et al., 1991; Bascom et al., 1992; Böhm et al., 2017). Potential tumor suppressor for lung cancer (Zhang M. et al., 2021)
Tetraspanin24	TSPAN24, CD151 molecule (Raph blood group), CD151 (PETA-3, RAPH, SFA-1	Low tissue specificity	Essential in kidney and skin morphogenesis (Karamatic Crew et al., 2004). Downregulation induces apoptosis in trophoblast cells in preeclampsia (Wang Z. et al., 2021). Marker for activated T cells (Perez et al., 2020)
Tetraspanin25	TSPAN25, CD53, MOX44	Blood, lymphoid tissue	Immune cell function regulator (Dunlock, 2020). Associated with tuberculosis (Omae et al., 2017; Jin et al., 2019). Leukocyte surface antigen (Angelisová et al., 1990; Horejsí and Vlcek, 1991)
Tetraspanin26	TSPAN26, CD37	Blood, lymphoid tissue	Potential biomarker in acute myeloid leukemia (Stilgenbauer et al., 2019; Zhang Q. et al., 2020). Leukocyte antigen (Classon et al., 1989; Horejsí and Vlcek, 1991). Associates with MHC class II glycoproteins (Angelisová et al., 1994)
Tetraspanin27	TSPAN27, CD82, IA4, KAI1, R2, ST6	Low tissue specificity	Suppresses migration in prostate cancer (Ichikawa et al., 1992; Dodla et al., 2020; Ma et al., 2020). Involved in pancreatic cancer (Liu X. et al., 2021). Regulation of oligodendrocyte progenitor cell myelination (Mela and Goldman, 2009)
Tetraspanin28	TSPAN28, CD81, TAPA-1, TAPA1	Low tissue specificity	Involved in lymphocyte cell membrane organization (Schaffer and Minguet, 2020). Knockout disrupts engraftment in acute lymphoblastic leukemia (Quagliano et al., 2020). Expressed on microglia (Reynolds and Mahajan, 2020)
Tetraspanin29	TSPAN29, CD9, BA2, MIC3, MRP-1, P24	Low tissue specificity	Regulates development of acute myeloid leukemia (Liu Y. et al., 2021). Associated with integrins (Torres-Gómez et al., 2021). Leukocyte surface protein (Horejsí and Vlcek, 1991). Expressed on microglia (Reynolds and Mahajan, 2020). Maintenance of the myelin sheet (Nakamura et al., 1996)
Tetraspanin30	TSPAN30, CD63, ME491, MLA1	Low tissue specificity	Negatively regulates hepatocellular carcinoma (Yu S. et al., 2021). Leukocyte surface glycoprotein (Horejsí and Vlcek, 1991). Involved in neural stem cell adhesion and migration (Lee et al., 2014)
Tetraspanin31	TSPAN31, SAS	Low tissue specificity	Suppresses cell proliferation of cervical cancer (Xia et al., 2020). Amplified in human sarcomas (Jankowski et al., 1995)
Tetraspanin32	TSPAN32, PHEMX, TSSC6	Blood, bone marrow, heart muscle, lymphoid tissue	Significantly reduced levels in CD4 T cells of multiple sclerosis patients (Lombardo et al., 2019)
Tetraspanin33	TSPAN33, MGC50844, Penumbra	Kidney	Regulates migration of human B lymphocytes (Navarro-Hernandez et al., 2020) and used as s marker of activated and malignant B cells (Luu et al., 2013). Regulates trafficking of the metalloprotease ADAM10 (Dornier et al., 2012). Highly expressed in erythroid progenitors (Heikens et al., 2007)

Data marked with (*) is obtained from the protein atlas database (proteinatlas.org). The “Tissue specificity” category is based on mRNA expression levels in the analyzed samples based on a combination of data from HPA, GTEX, and FANTOM5 obtained from proteinatlas.org.

## The Structure of Tetraspanins

**FIGURE 1:**
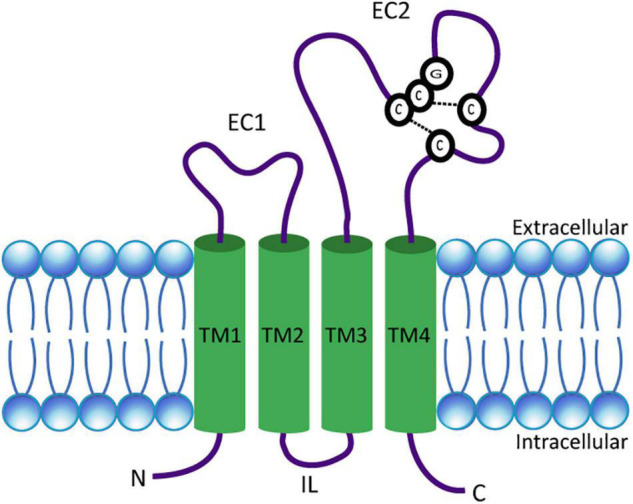
Schematic presentation of a tetraspanin. The model depicts the conserved structural features of tetraspanins. EC1 and EC2 represent the short and large extracellular domains, respectively. Transmembrane domains TM1–TM4 and the CCG motif along with disulfide bonds between two pairs of cysteine residues in EC2 are shown. N- and C-termini as well as the intracellular loop (IL) are located in the cytosol.

Tetraspanins are small integral membrane proteins that range in size from 204 (Tspan13) to 355 (Tspan10) amino acids (Charrin et al., 2009) and have a molecular weight of 20–30 kDa (Bassani and Cingolani, 2012). Topologically, tetraspanins possess four TMs, where the first two (TM1 and TM2) are linked by a small extracellular loop (EC1) of 20–28 amino acids and the last two (TM3 and TM4) are connected by a large extracellular loop (EC2) of 76–131 amino acids (Berditchevski, 2001; Huang et al., 2005; Lazo, 2007). TM2 and TM3 are joined by a very short intracellular loop (IL) (Figure 1). In addition, tetraspanins comprise short cytosolic N- and C-termini (Huang et al., 2005; Seigneuret, 2006). Tetraspanins are distinguished from other proteins with four TMs by several distinct features. These include the unequal size of the two extracellular loops, the presence of two to four cysteine pairs, and a CCG motif (Cys-Cys-Gly sequence) in EC2 (Figure 1). In addition, over 50% of tetraspanins carry a PxxCC (Pro-x-x-Cys-Cys) motif, where x can be any amino acid (Berditchevski, 2001; Huang et al., 2005; Seigneuret, 2006). The cysteine pairs in EC2 play an essential role for the correct folding of the domain by forming disulfide bridges (Berditchevski, 2001). Furthermore, a highly conserved subfamily of tetraspanins known as TspanC8, comprising Tspan5, Tspan10, Tspan14, Tspan15, Tspan17, and Tspan33, is characterized by their unique property of possessing eight cysteines within their EC2 (Boucheix and Rubinstein, 2001; Huang et al., 2005). Most tetraspanins are potentially N-glycosylated in EC2 (Maecker et al., 1997). Tetraspanins known to be un-glycosylated are Tspan28 (CD81) and Tspan12 (Net-2), while Tspan29 (CD9) contains an N-glycosylation site in EC1 (Boucheix and Rubinstein, 2001). Presumably, most tetraspanins are also S-acylated. For example, palmitoylation has been demonstrated in three independent studies for each tetraspanin tested (Seehafer et al., 1988; Berditchevski et al., 2002; Charrin et al., 2002; Yang et al., 2002; Stipp et al., 2003). In addition, ubiquitination has already been demonstrated for Tspan6 (Wang et al., 2012), Tspan24 (CD151), Tspan27 (CD82) (Tsai et al., 2007), and Tspan28 (CD81) (Lineberry et al., 2008; Charrin et al., 2009; Termini and Gillette, 2017).

## Physiological Functions of Tetraspanins

In addition to the research dedicated to uncovering all of the structural peculiarities of this protein family, a major effort has been made over the last 20 years to shed more light on the involvement of these proteins in various physiological functions in the body. Many tetraspanins are involved in the process of cell development, activation, growth regulation, and motility. It has been shown that Tspan1 significantly reduces cell migration, tissue invasion, and increases apoptosis of human pancreatic cancer cells (Tian et al., 2018). Furthermore, the expression of Tspan3, Tspan4, and Tspan7 in cells with high migratory potential suggests a role in the regulation of migration processes (Kashef et al., 2013). Down-regulation of Tspan8 inhibits the proliferation and migration of colorectal cancer cells, while over-expression promotes the opposite effects (Zhang et al., 2019). Overexpression of Tspan9 significantly inhibits the proliferation, migration, and invasion of human gastric cancer cells (Li et al., 2016). Direction of bone matrix organization orthogonal to osteoblast alignment is controlled by Tspan11-mediated focal adhesion assembly (Nakanishi et al., 2019).

Another context in which tetraspanins are involved, is the immune system and regulation of inflammatory pathways. Tspan18 knockout mice have reduced thrombus size in a deep vein thrombosis model, and reduced platelet deposition in the microcirculation following myocardial ischemia-reperfusion injury (Noy et al., 2019). Tspan26 is a B-cell surface antigen widely expressed on mature B cells, and it is involved in immune regulation and tumor suppression (Xu-Monette et al., 2016). Tspan28 (CD81) knockout cells show impaired viral DNA replication and produce greatly diminished viral titers in Herpes simplex virus 1 infection in a neuroblastoma cell model (Benayas et al., 2020).

Since Tspan28, Tspan29, and Tspan30 are abundantly expressed on extracellular vesicles categorized as exosomes, they are also used as exosomal markers (Lai et al., 2010; Oksvold et al., 2015). Tspan31 is a critical regulator of transduction of survival and apoptotic signals in hepatocellular carcinoma cells (Wang et al., 2018). Two tetraspanins (Tspan20 and Tspan21), better known as uroplakins (UPKs), are involved in normal bladder epithelial physiology. Tspan20, also known as uroplakin 1B (UPK1B), promotes the invasion and metastasis of bladder cancer via regulation of the Wnt/β-catenin pathway (Wang et al., 2018; Zhang Z. Y. et al., 2020), while the reduced expression of Tspan21 (UPK1A) might play a role in the progression of gastric cancer (Zheng et al., 2014).

The standardized tetraspanin nomenclature (Charrin et al., 2009) utilizes a simple numbering system from Tspan1 to Tspan33. However, in certain research fields other names for some of the tetraspanin proteins are more frequently used. A summary of all human tetraspanins including their alternative names, tissue-specific RNA expression, and their reported involvement in different physiological or pathophysiological processes is listed in Table 1.

## Tetraspanins in the Central Nervous System

One of the first evidences of tetraspanins playing an important role in the CNS came from research on the *Drosophila melanogaster* tetraspanin late bloomer (lbl) in the middle of the 1990s. It was observed that in fly mutants lacking this protein, adjacent motoneurons showed increased ectopic sprouting, and synapse formation was delayed. These results, which indicate that lbl promotes the formation of motor neuron synapses in the fruit fly, suggest that other tetraspanins may also be involved in this process and that a similar mechanism may also be found in mammals (Kopczynski et al., 1996). Furthermore, RNA *in situ* analyses of all tetraspanin family genes found in *Drosophila* revealed that most tetraspanins are either specifically expressed in the nervous system or intestines or have ubiquitously low levels of expression. In addition, other studies suggested that the tetraspanins are not critical for neurite growth but that their absence delays the formation of the final synaptic contact points of motor neuron axons. Moreover, the tetraspanins expressed by motor neurons showed partial compensation for the role of deleted lbl (Fradkin et al., 2002).

Following these studies in *Drosophila*, numerous research efforts in the following years focused on demonstrating the importance and involvement of tetraspanins in various processes in the CNS. Since the tetraspanins are referred to as “molecular organizers,” it became clear that certain tetraspanins playing an important role in regulating ADAM10 (A disintegrin and metalloproteinase 10), are involved in the Notch signaling pathway, and are essential for photoreceptor function.

### ADAM10 Regulation by Tetraspanins

Tetraspanins have been reported to regulate metalloproteases. Particularly important for the nervous system is ADAM10 also known as Kuzbanian in *Drosophila*, which works as “molecular scissors” and is responsible for cleaving the extracellular portion of dozens of transmembrane proteins including amyloid precursor protein (APP), notch, and cadherin (Hartmann et al., 2002; Reiss et al., 2005; Kuhn et al., 2010; Dornier et al., 2012; Groot et al., 2014; Seipold and Saftig, 2016). It has been shown that Tspan3 acts as a stabilizing factor of active ADAM10, APP, and the γ-secretase complex at the plasma membrane and Tspan12 and Tspan15 may be involved in Alzheimer’s disease physiopathology (Seipold et al., 2017). It was found that all members of the TspanC8 subfamily are directly involved in the regulation of ADAM10 exit from the ER and play an important role in its enzymatic maturation by promoting its trafficking to the cell surface (Dornier et al., 2012). TspanC8s, indeed, differentially regulate ADAM10 membrane compartmentalization through atypical association. Four TspanC8 members, in particular Tspan5, Tspan14, Tspan15, and Tspan33, increase ADAM10 expression at the cell surface (Dornier et al., 2012). Mass spectrometric analysis suggested a reduced interaction of ADAM10 with other components of the tetraspanin web in Tspan15-expressing cells. By contrast, ADAM10 association with other members of the C8 tetraspanin subfamily was increased in Tspan5-transfected cells (Jouannet et al., 2016). Recently, with the help of the first monoclonal antibodies generated against Tspan15, it was shown that ADAM10 and Tspan15 exist together at the cell surface and that ADAM10 is required for Tspan15 expression in cell lines (Koo et al., 2020). This suggests that the direct interaction of Tspan15 with ADAM10 makes it crucial for the ADAM10 scissor complex (Koo et al., 2020). In an independent study, mass spectrometric analysis revealed Tspan12 as a partner for ADAM10 that is involved in its maturation and increases ADAM10-mediated cleavage of the APP (Xu et al., 2009). Tspan15 was identified as the only member of the TspanC8 family involved in the promotion of ADAM10-dependent N-cadherin cleavage when overexpressed in human embryonic kidney cells (Noy et al., 2016). Some integrin–tetraspanin interactions facilitate maturation and cell surface expression of the integrin receptors. Since many tetraspanins contain PDZ domain-binding motifs, it is possible that some of them serve as linkers between integrins and intracellular PDZ scaffolding proteins or signal transduction molecules (Bassani and Cingolani, 2012).

### Role of Tetraspanins in Notch Signaling

Notch has been established as one of the major substrates of the ADAM10 protease (Groot et al., 2014). Several studies have suggested that Notch has a crucial role in the synaptic plasticity of the mammalian brain (Costa et al., 2003, 2005; Wang et al., 2004). Depending on the level of receptor activation, Notch might decrease or increase long-term potentiation (LTP) (Wang et al., 2004; Dahlhaus et al., 2008). Human Tspan5 and Tspan33, the orthologs of *Caenorhabditis elegans* TSP-12, appear to facilitate γ-secretase-mediated cleavage and thus promote Notch activity (Dunn et al., 2010). Further investigation revealed that other members of TspanC8, Tspan5, and Tspan14, also appear to be promoters of ligand-induced Notch activity in U2OS and HeLa cells, while Tspan15 has the opposite effect (Dornier et al., 2012; Jouannet et al., 2016). The latter observation explains why the knockdown of Tspan15 enhances Notch activation (Jouannet et al., 2016).

### The Tspan22 (peripherin 2) Connection to Photoreceptors

Human Tspan22, also known as peripherin 2 (PRPH2) or retinal degeneration slow (RDS), is located in the retina and abundantly expressed in the outer segment (OS) of photoreceptors (Molday and Molday, 1987; Milstein et al., 2020). PRPH2 is thought to be essential for the vision and morphogenesis of the OS, which consists of hundreds of disc membranes mainly responsible for the conversion of light signals into electrical signals. Membranous discs in OS undergo constant renewal (Young, 1967). In this regard, PRPH2 in a complex with rom-1 (rod outer segment membrane protein 1, which is another related tetraspanin) is required for the regulation of morphogenesis and structural integrity of the OS (Stuck et al., 2016; Murru et al., 2018). It was found that any gene-level mutation or defect in PRPH2 leads to a broad range of progressive retinal degeneration events in humans (Kohl et al., 1998), affecting mainly either rod or cone cell photoreceptors (Stuck et al., 2016). PRPH2-deficient mice show disrupted photoreceptor morphogenesis (Sanyal et al., 1980). In addition, PRPH2 serves as a molecular bridge between rhodopsin and the rod cyclic nucleotide-gated (CNG) channel complex in rod OS (Becirovic et al., 2014).

## Tetraspanins in the Brain

**FIGURE 2:**
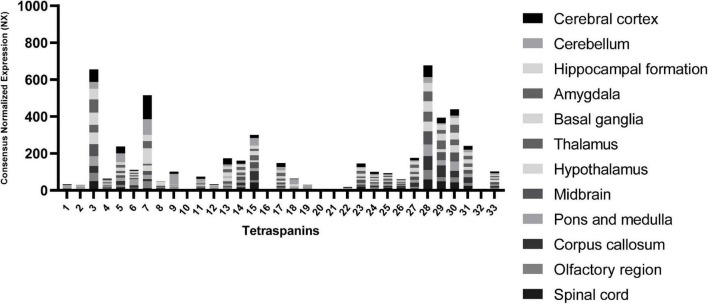
RNA expression levels of tetraspanins in different regions of the brain. Consensus Normalized eXpression (NX) levels shown are created by combining the data from three transcriptomics datasets (HPA, GTEx, and FANTOM5) using normalization pipeline. Each bar contains combined data of all brain regions indicated. Data used to compose the graph obtained from the Human Protein Atlas database (proteinatlas.org).

To point out other tetraspanins that may play important roles in the CNS, we extracted RNA expression level data in different brain regions from “The Human Protein Atlas” database (Uhlén et al., 2015) in Figure 2.

The tetraspanin with the strongest expression in the brain tissue is Tspan7 (Table 1), which also shows the strongest localization in cerebral cortex in comparison to other regions of the brain (Figure 2). Tspan7 was characterized as a key player in the morphological and functional maturation of glutamatergic synapses (Bassani and Passafaro, 2012). Tspan3 stands out as having one of the highest RNA expression levels in the brain among all tetraspanins (Figure 2).

Interestingly, the tetraspanins Tspan28, Tspan29, and Tspan30 exhibit overall high levels of RNA expression. Tspan29 and Tspan28 are shown to be expressed on microglial cells (Reynolds and Mahajan, 2020). Tspan29, more commonly called CD9, is involved in neurite outgrowth and cell migration, and it is associated with the α6β1 integrin and the neural adhesion molecule L1 in mouse brain (Schmidt et al., 1996). Tspan29 was also detected in human adult nervous tissue, and its expression is correlated with myelination where it may interact with the extracellular matrix and participate in the maintenance of the myelin sheath (Nakamura et al., 1996). It also enhances myelin membrane adhesion to extracellular matrices at very late stages of development (Kagawa et al., 1997). Furthermore, Tspan29 plays a role in glial cells in pathological conditions such as the devastating transmissible spongiform encephalopathy (TSE) (Doh-Ura et al., 2000).

Involvement of Tspan13 in modulating the coupling between the voltage sensor activation and pore opening of voltage-gated Ca^2+^ channels (encoded by the caveolin genes, abbreviated as CAV) was confirmed where Tspan13 has been identified as an interaction partner of the α1 subunit of N-type CaV2.2 (Mallmann et al., 2013).

Tspan27 (CD82) is a critical molecule in the regulation of oligodendrocyte progenitor cell migration and myelination (Mela and Goldman, 2009). Downregulation of this molecule in oligodendrocytes inhibits differentiation, reduces myelin protein accumulation, and leads to regression to less mature stages (Mela and Goldman, 2013). In addition, there is evidence that the CD82-TRPM7-Numb pathway is associated with age-related synapse/memory impairments (Zhao et al., 2020). Further, TIMP-1 (tissue inhibitor of metalloproteinase-1) has been identified as a potent key regulator of Tspan30 (CD63) and β1-integrin-mediated signaling that regulates human neural stem cell adhesion and migration (Lee et al., 2014).

## Tetraspanins at Glutamatergic Synapses

Ionotropic glutamate receptors (iGluRs) are ligand-gated cation selective ion channels that can be activated by the brain’s major excitatory neurotransmitter L-glutamate. The family of iGluRs can be further subdivided into kainate (KA), N-methyl-D-aspartate (NMDA), α-amino-3-hydroxy-5-methyl-4-isoxazole propionate (AMPA) and Delta receptors (Hollmann and Heinemann, 1994; Sobolevsky et al., 2009; Traynelis et al., 2010). AMPARs are typically located at the post-synapse and mediate the majority of rapid excitatory neurotransmission (Gasic and Hollmann, 1992). They play a critical role in mechanisms such as synaptic plasticity and are associated with various neurodegenerative and neuropsychiatric disorders (Palmer et al., 2005). The AMPA receptor (AMPAR) subfamily consists of a total of four members (GluA1–GluA4) and they are functional as homomeric and heteromeric tetramers (Sager et al., 2009). Many different auxiliary subunits have been discovered, which influence AMPAR function in the CNS with respect to trafficking and gating. The most prominent and intensively studied auxiliary proteins are the so-called transmembrane AMPAR modulatory proteins (TARPs), of which there are six members in total (γ2, γ3, γ4, γ5, γ7, and γ8) (Tomita, 2010), with γ2 being the most extensively member studied to date. For example, γ2 promotes trafficking of AMPARs to the plasma membrane and influences their biophysical functions (Tomita, 2010). Thus, γ2 is able to slow receptor deactivation and at the same time strongly reduces receptor desensitization accompanied by a faster recovery from desensitization (Priel et al., 2005; Tomita, 2010; Ben-Yaacov et al., 2017). In addition, γ2 increases agonist affinity and influences receptor gating by increasing channel conductance and opening probability (Tomita, 2010; Ben-Yaacov et al., 2017).

Research over the last 20 years has revealed many interacting proteins that play a key role in the regulation of glutamate receptor trafficking and functions, which in turn leads to stabilization and strengthening of synaptic plasticity. Major directly interacting proteins of AMPARs include GRIP1 (glutamate receptor-interacting proteins 1) (Osten et al., 2000) and PICK1 (protein interacting with C kinase 1) (Xia et al., 1999), required for either insertion of AMPARs at the synaptic surface (Osten et al., 2000) or removal from the synaptic membrane (Braithwaite et al., 2002) as well as for the regulation of spine morphology.

Tspan6, associated with the neurological condition EFMR (epilepsy female-restricted with mental retardation, now referred to as intellectual disability), has been identified as a novel regulator of hippocampal synaptic transmission and LTP, with a key role in synapse development and AMPAR trafficking (Depienne et al., 2011; Vincent et al., 2012; Salas et al., 2017). It was speculated that the increased synaptic transmission observed by some authors was caused by an increased response to glutamate (Salas et al., 2017). However, no altered expression of synaptic proteins was detected (Salas et al., 2017). In fact, the expression of postsynaptic density protein 95 (PSD-95), AMPARs, NMDARs, and metabotropic glutamate receptors (mGluRs) was unchanged in the postsynaptic membrane (Salas et al., 2017). This observation applies equally to intracellular receptors, which are receptors that are not located in the plasma membrane but in intracellular compartments of the neuron, such as the ER or endosomes, and cell-surface receptors as well as to synaptically and non-synaptically localized GluA1-containing AMPARs (Salas et al., 2017). Moreover, no change in phosphorylation could be detected for the GluA1 subunit (Salas et al., 2017). The authors combine their seemingly contradictory results of an observed increase in basal synaptic transmission in combination with reduced LTP in the hypothesis of “occluded” LTP (Salas et al., 2017). This hypothesis posits that sustained, increased basal synaptic transmission inhibits the induction of LTP because further excitatory stimulation is not possible anymore. It has also been speculated that Tspan6 alters the biophysical properties of AMPARs, possibly in a TARP-like manner (Salas et al., 2017), so a direct interaction between Tspan6 and AMPARs may be possible.

Tspan7, which is highly homologous to Tspan6, is associated with the neurological disease X-linked intellectual disability (Zemni et al., 2000; Abidi et al., 2002; Bassani et al., 2012). Mutation of the TM4SF2 gene (=Tspan7) is a cause of a severe intellectual disability and cognitive impairment. The mutation is connected with alterations in AMPAR expression levels, which cause changes in excitatory synapse structure and function (Murru et al., 2017). The ampakine CX516 has been shown to have positive effects on Tspan7 knockout mice, rescuing the intellectual disability phenotype, suggesting pharmacological modulation of AMPARs as a potential therapeutic target (Murru et al., 2017).

**FIGURE 3:**
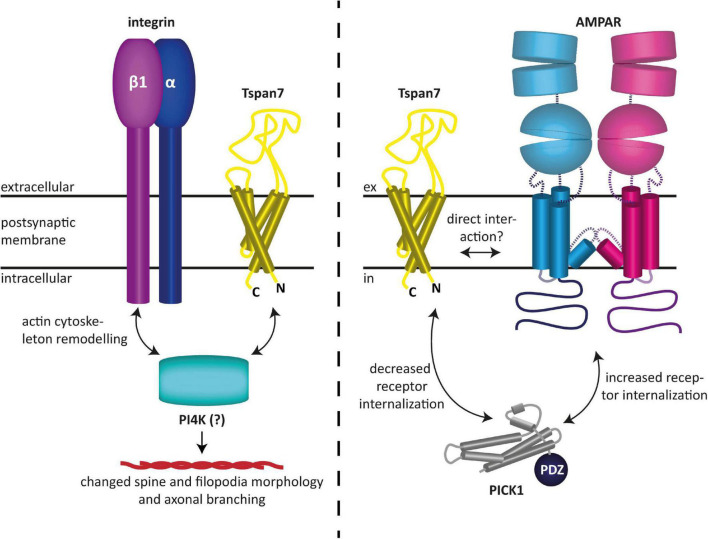
Reported functions of Tspan7 in neurons. (Left) Tspan7 influences neuronal morphogenesis by regulating filopodia density and dendritic spine morphology. This is presumably mediated by interactions with integrin β1 and/or PI4K and ultimately results in remodeling of the actin cytoskeleton (Bassani et al., 2012). (Right) Tspan7 regulates synaptic function at glutamatergic synapses. Interaction of Tspan7 with PICK1 attenuates the internalization of AMPARs, which is typically mediated by the interaction of the latter with PICK1 (Perez et al., 2001). This subsequently leads to an increased availability of AMPARs at the postsynaptic membrane and thus to an enhanced excitability of the postsynaptic neuron. In this process, Tspan7 interacts with PICK1 via its C-terminus (Bassani et al., 2012). The figures are based on the illustrations of Bassani and Passafaro (2012) and Perot and Ménager (2020).

Tspan7 has also been found to modulate glutamatergic synaptic transmission as well as synaptic plasticity (Bassani et al., 2012; Salas et al., 2017). In particular, it was shown to promote dendritic spine formation (Bassani et al., 2012). Effectively, Tspan7 influences neuronal morphogenesis by regulating filopodia density and dendritic spine morphology (Bassani et al., 2012). This is presumably mediated by interactions with integrin β1 and/or phosphatidylinositol 4-kinase (PI4K), and ultimately results in remodeling of the actin cytoskeleton, as speculated by the authors (Bassani et al., 2012). The authors furthermore demonstrated that Tspan7 interacts with the PDZ domain of PICK1 to regulate AMPAR trafficking and hippocampal spine development *in vitro* (Bassani et al., 2012). It was shown that the interaction of Tspan7 with PICK1 attenuates the internalization of AMPARs, which is typically mediated by the interaction of the latter with PICK1 (Perez et al., 2001). This subsequently leads to an increased availability of AMPARs at the postsynaptic membrane and thus to an enhanced excitability of the postsynaptic neuron (Bassani et al., 2012). Because co-immunoprecipitation experiments have shown that the C-terminus of Tspan7 knocks down PICK1 and β1 integrin as well as the two AMPAR subunits GluA2 and GluA3, a macromolecular complex between the aforementioned proteins, in which Tspan7 serves as an organizer, has been hypothesized (Bassani et al., 2012). However, a direct modulation of AMPARs by Tspan7, similar to what has been proposed for Tspan6, appears equally plausible, as it could also explain the findings described in Bassani et al. (2012). In fact, like the prototypical TARP γ2, Tspan7 may preferentially interact with the GluA2 subunit, as it colocalizes with it most predominantly (Bassani et al., 2012), and hence could be responsible for the maintenance of normal synaptic plasticity. The fact that in the co-immunoprecipitation experiments Tspan7 pulled down two AMPAR subunits, namely GluA2 and GluA3, in addition to other proteins, may also indicate a direct interaction between the aforementioned proteins, although none of the proteomics studies published to date have found tetraspanins among the AMPAR-associated proteins discovered with these methods (Schwenk et al., 2012, 2014; Li et al., 2013; Chen et al., 2014; Bettler and Fakler, 2017). However, this does not necessarily mean that tetraspanins do not interact with AMPARs, as these studies rely on the specific properties of certain mild detergents, which may not, however, be suitable for maintaining the specific interactions of tetraspanins. An overview of the functions of Tspan7 in neurons described here and found by Bassani et al. (2012) is shown in Figure 3.

## Structural Homologies Between Tetraspanins and Known Ampa Receptor Modulators

**FIGURE 4:**
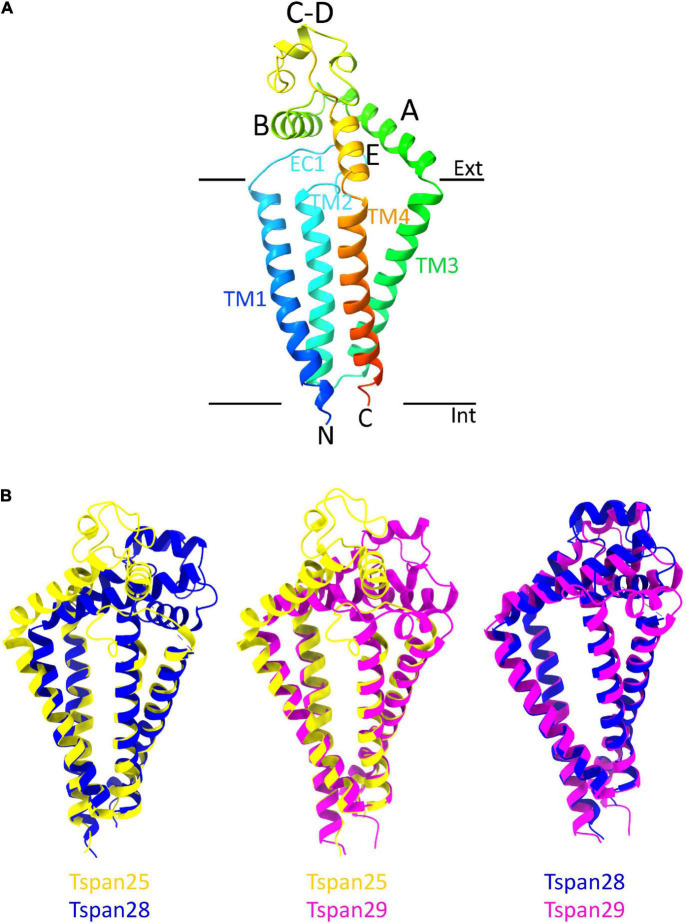
Overall structure of a tetraspanin **(A)**. Helices A–E are indicated in the EC2 domain between TM3 and TM4. Helices A, B, and E represent conserved regions, while helices C–D are variable among tetraspanins. Superposition of all previously resolved structures of tetraspanins **(B)**. Shown is the overlay of Tspan25 (pdb model 6WVG in yellow) with Tspan28 (pdb model 5TCX in blue) and of Tspan25 (yellow) with Tspan29 (pdb model 6K4J in magenta) as well as of Tspan28 (blue) with Tspan29 (magenta).

From the entire tetraspanin family, only the full-length structures of Tspan28 (CD81) (Zimmerman et al., 2016; Yang et al., 2020; Susa et al., 2021), Tspan29 (CD9) (Umeda et al., 2020), and Tspan25 (CD53) (Yang et al., 2020) have been resolved to date, and the structures were found to be highly similar. Thus, the four TMs of Tspan25, Tspan28, and Tspan29 each form an ice cream cone-shaped structure with two TM pairs forming the sides of this structure (Figure 4B; Zimmerman et al., 2016; Umeda et al., 2020; Yang et al., 2020). The TM domains of all solved tetraspanins structures are virtually superimposable (Figure 4B), with Tspan25 in the open conformation, without bound lipid, adopting the same structure as Tspan28 and Tspan29 in the closed form with bound cholesterol (Yang et al., 2020). The EC2 domains of the beforementioned tetraspanins can be generally described as mushroom-like structures that have a total of five helices A to E. Here, helices A and E form the fungal stem, while helix B forms the fungal head together with the variable C–D region (Figure 4A) (Zimmerman et al., 2016; Umeda et al., 2020; Yang et al., 2020). The general mushroom shape is visible in all resolved structures, whereas slightly larger structural variations are apparent in the C–D region (Figure 4B).

The EC2 domain is thought to be responsible for the partnering of the respective tetraspanins with other proteins, a property facilitated by its sequence and structural variability (Zimmerman et al., 2016; Umeda et al., 2020; Yang et al., 2020). The shorter EC1 domain appears disordered in most structural resolutions representing the closed state of the respective molecule, suggesting a high flexibility of this domain (Yang et al., 2020; Susa et al., 2021). Based on the recently resolved structures of the open state, in which the EC1 domain is well visible, it can be assumed that EC1 stabilizes the open conformation through interaction with EC2 (Yang et al., 2020; Susa et al., 2021).

**FIGURE 5:**
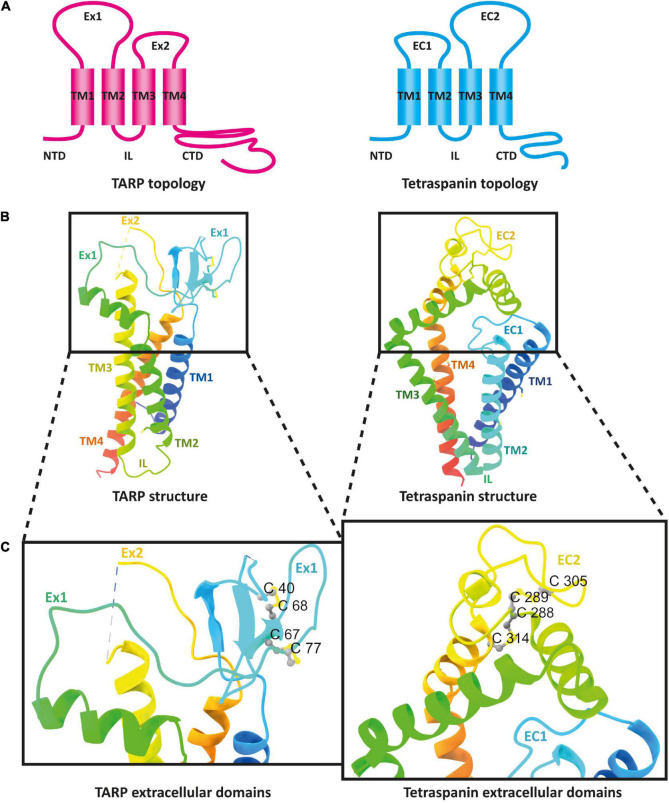
The architecture of transmembrane AMPAR regulatory proteins (TARPs) and tetraspanins (TSPANs). **(A)** Overall topology of TARPs (left) in comparison to tetraspanins (right). **(B)** Ribbon diagrams, rainbow-colored (from N-terminus in blue to C-terminus in red), representing the structures of TARPs (left) based on the structure of human TARP γ2, originally named stargazin and resolved by cryo-EM (pdb model 6DLZ, published by Twomey et al., 2018), and human Tspan25 (right), also known as CD53, resolved by crystallization and X-ray diffraction (pdb model 6WVG, published by Yang et al., 2020). Cysteine residues and disulfide bridges are presented as yellow heteroatoms and sticks, respectively. Distinct domains are labeled. Black boxes indicate the areas of TARPs and tetraspanins enlarged in panel **(C)**. Cysteine residues forming disulfide bridges in the big loops are labeled (one-letter amino acid code with number of each residue) and presented in ball and stick style with sulfur and carbon shown in yellow and gray, respectively.

Looking at the membrane topology, the close similarity between TARPs and tetraspanins is obvious (Figure 5A). Both proteins have four TM domains, although these adopt a more conical form in the previously resolved structures of tetraspanins compared with those of TARPs (Figure 5B). In the extracellular domain, both protein families are topologically very similar. Both feature a large extracellular loop (Ex1 in TARPs and EC2 in tetraspanins) and a small loop (Ex2 in TARPs and EC1 in tetraspanins). These loops are folded differently in the structures resolved so far. The small Ex2 domain in TARPs contains a β-sheet and a loop according to the available resolved structures, which unfortunately have been fully resolved so far only for TARP γ5 (pdb model 7RZ5) (Klykov et al., 2021). In tetraspanins, the small EC1 loop either does not contain a secondary structure element, i.e., comprises exclusively a loop, as in Tspan25 (pdb model 6WVG) (Yang et al., 2020), or EC1 contains an α-helix along with a loop, as in Tspan28 (pdb model 7JIC) (Susa et al., 2021) and Tspan29 (pdb model 6K4J) (Umeda et al., 2020). The small loops are overall shorter in tetraspanins than in TARPs and presumably serve to stabilize the larger EC2 loop (Yang et al., 2020). For TARPs, both loops are thought to have a receptor-modulating function, although there is no consensus so far, as different observations have been reported (Twomey et al., 2016; Riva et al., 2017; Herguedas et al., 2019). Remarkable differences exist in the formation of secondary structural elements. The Ex1 loop forms one α-helix and four β-strands in TARPs, whereas the large loop in the tetraspanins exclusively forms α-helical elements (Figures 5B,C). The formation of disulfide bridges in the large EC2 domain of tetraspanins is likely important for proper folding. However, TARPs also have several cysteine residues in their large Ex1 loop that form disulfide bridges (Figure 5C).

**FIGURE 6:**
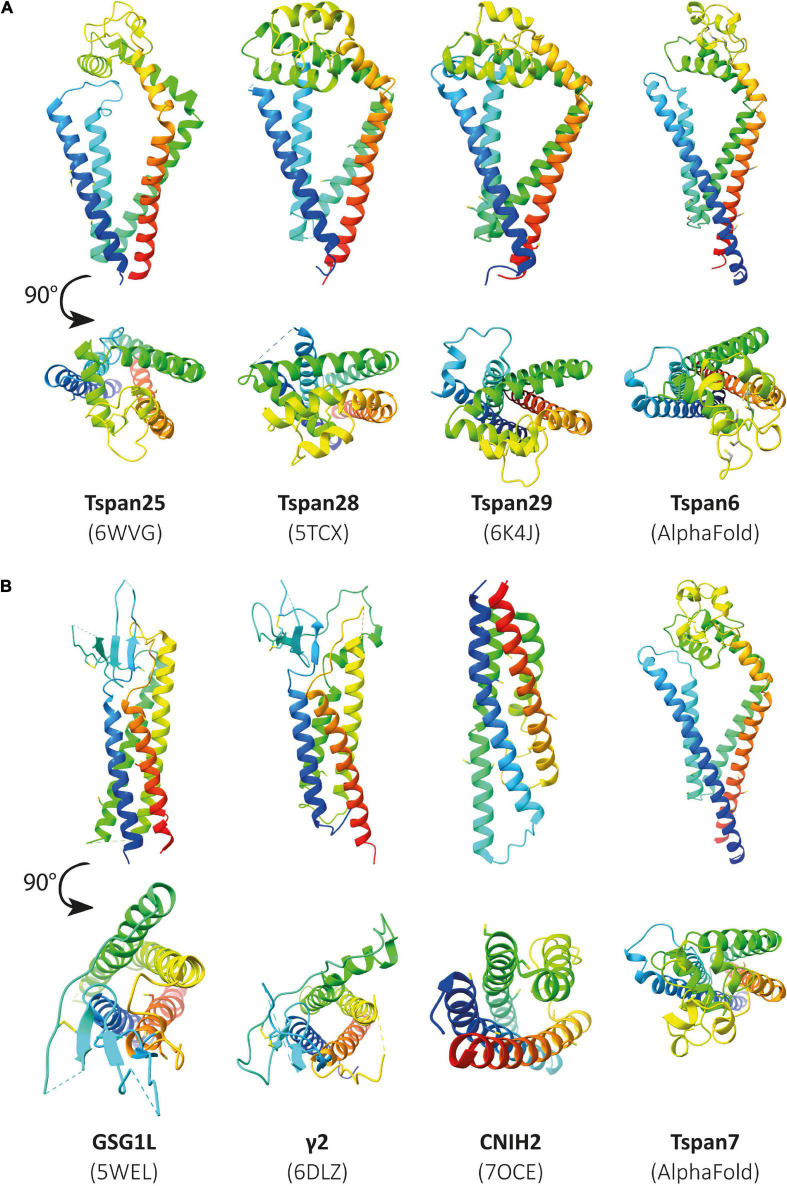
Illustration of previously resolved and predicted tetraspanin structures in comparison to resolved structures of known AMPAR auxiliary subunits. Ribbon diagrams rainbow-colored (from N-terminus in blue to C-terminus in red) showing the structures of the chosen proteins from *Homo sapiens*, displayed with different views, one parallel to the membrane (upper) and another from the extracellular side rotated by a 90° angle (lower). In panel **(A)** the following structures are depicted (from left to right): Tspan25 (pdb model 6WVG), Tspan28 (pdb model 5TCX), Tspan29 (pdb model 6K4J), and Tspan6 (AlphaFold database). Panel **(B)** shows the structures of GSG1L (pdb model 5WEL), γ2 (pdb model 6DLZ), CNIH2 (pdb model 7OCE), and Tspan7 (AlphaFold database) from left to right.

Unfortunately, the structures resolved to date do not include tetraspanins associated with CNS functions such as Tspan6 or Tspan7. So far, the structure of many different proteins, identified as auxiliary subunits of AMPARs, has been elucidated. These include structures of the TARPs γ2 (Twomey et al., 2016), γ5 (Klykov et al., 2021), and γ8 (Herguedas et al., 2019), GSG1L (germ cell-specific gene 1-like protein) (Chen and Gouaux, 2019), and CNIH3 (cornichon family AMPA receptor auxiliary protein 3) (Nakagawa, 2019) as well as cornichon family AMPA receptor auxiliary protein 2 (CNIH2) (Zhang D. et al., 2021). If some tetraspanins do indeed interact with or modulate the function of AMPARs, as has been postulated, it may be assumed that they are structurally similar to already known AMPAR auxiliary subunits. In Figure 6, already known structures of selected AMPAR auxiliary subunits are shown in comparison to the previously resolved structures of Tspan25, Tspan28, and Tspan29. In the absence of solved structures for Tspan6 and Tspan7, which serve essential roles in the CNS (Bassani et al., 2012; Salas et al., 2017), predicted structures of Tspan6 and Tspan7 were used which have been mapped using the predictions of DeepMind’s new artificial intelligence called AlphaFold (Jumper et al., 2021; Tunyasuvunakool et al., 2021).

**FIGURE 7:**
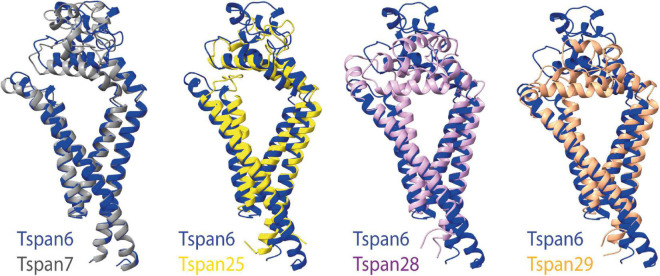
Superposition of selected tetraspanin structures with the predicted structure of Tspan6. The left side shows the superposition of the structures of Tspan6 (blue) and Tspan7 (gray) predicted by AlphaFold; both tetraspanins are known to influence glutamatergic synapses. For comparison, the overlays of the structure of Tspan6 with already known resolved structures of tetraspanins are shown on the right (from left to right): overlay of Tspan6 with Tspan25 (yellow) (PDB model 6WVG) as well as with Tspan28 (pale pink) (PDB model 5TCX), and with Tspan29 (orange) (PDB model 6K4J).

Figure 6A (three structures on the left) shows all previously known tetraspanin structures (Tspan25 or CD53, Tspan28 or CD81, and Tspan29 or CD9) in two views: parallel to the membrane, and in a birds-eye view from above the membrane. Both views are compared to three selected AMPAR auxiliary subunits (Figure 6B): GSG1L, TARP γ2, and CNIH2. GSG1L and γ2 are structurally strikingly similar, whereas CNIH2 adopts a different fold. The structures predicted by AlphaFold for Tspan6 and Tspan7 (Figures 6A,B, two structures on the right), for each of which an essential function in the CNS has already been demonstrated (Bassani et al., 2012; Murru et al., 2017; Salas et al., 2017), are shown in comparison to γ2 (Figure 6B, center). Both are predicted to have a similar conformation, which differs markedly from that of the previously resolved tetraspanin structures (see Figure 7). Tspan6 (blue) and Tspan7 (gray) are generally less conical, their first as well as last TM domains are much longer, and the large extracellular loop, while also forming α-helices, has a significantly different spatial structure by comparison. Of all the tetraspanin structures resolved so far, Tspan25 (yellow) has the most similar structure to Tspan6, whereas Tspan28 (pink) and Tspan29 (orange) show stronger structural deviations from Tspan6.

**FIGURE 8:**
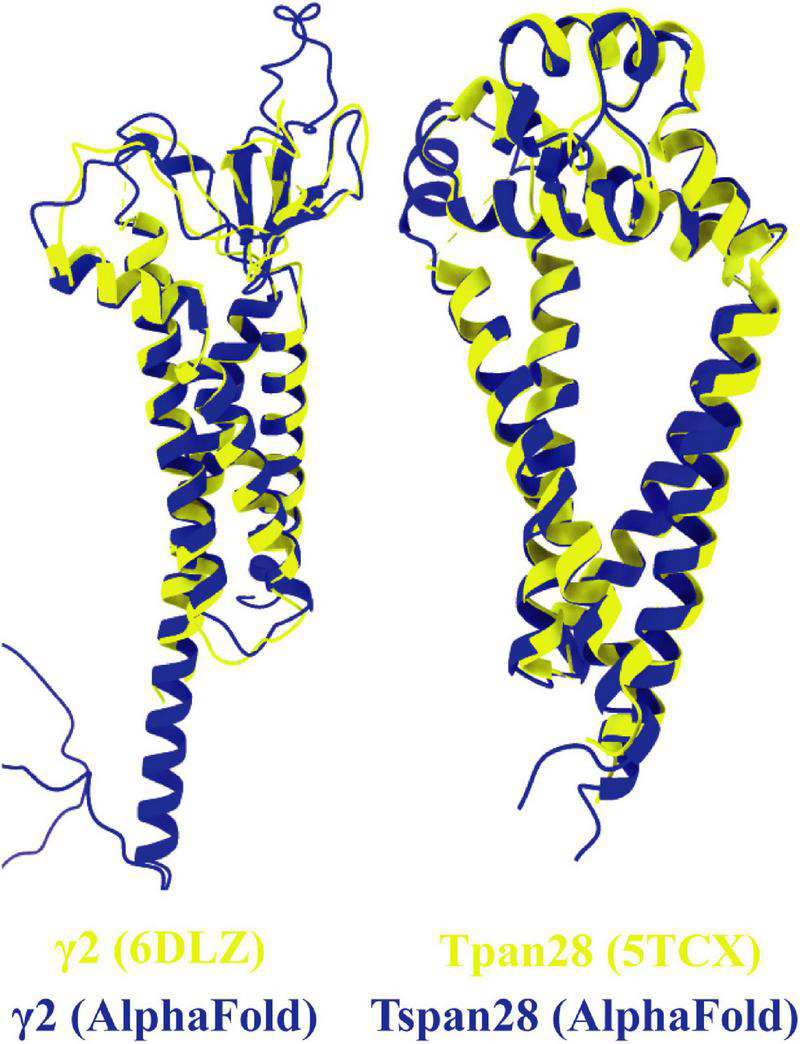
Overlays of predicted and resolved structures. To obtain an impression of the accuracy and reliability of the structures predicted by AlphaFold, the corresponding pdb files of the solved structures of γ2 [(left) pdb model 6DLZ, colored in yellow] and Tspan28 [(right) pdb model 5TCX, colored in yellow] were superimposed on the respective AlphaFold predictions (colored in blue).

To compare the prediction of AlphaFold with experimentally resolved protein structures, the prediction for γ2 was superimposed on a structure of γ2 elucidated by cryo-EM (Figure 8, left). Overall, the two structures from *Homo sapiens* match well. As expected, the motile parts of the loops diverge slightly as they represent flexible elements. Somewhat unexpectedly, the TM4 of γ2 is predicted by AlphaFold to be much longer than was determined by experimental analysis. The predicted structure for Tspan28 (AlphaFold), on the other hand, overlaps almost perfectly with the resolved structure (Figure 8, right), confirming AlphaFold as a trustworthy, powerful tool for *in silico* analysis of the proteins studied here.

**FIGURE 9:**
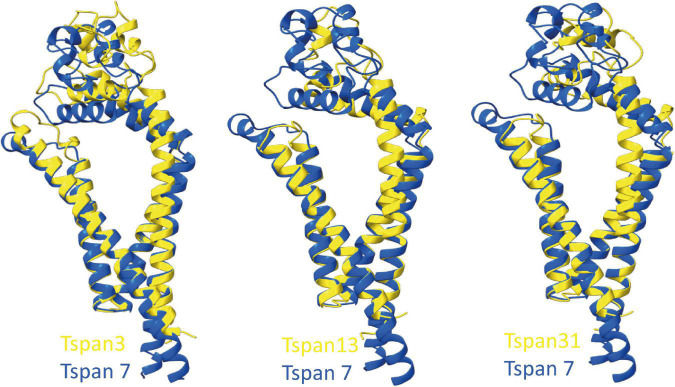
Superimposition of AlphaFold-predicted structures of Tspan3, Tspan13, and Tspan31 with Tspan7 using ChimeraX. Aligned structures of Tspan3, Tspan13, and Tspan31 (yellow) and Tspan7 (blue). RMSD values for Tspan3/7, Tspan13/7, and Tspan31/7 are 0.9, 0.829, and 0.854 Å, respectively.

**FIGURE 10:**
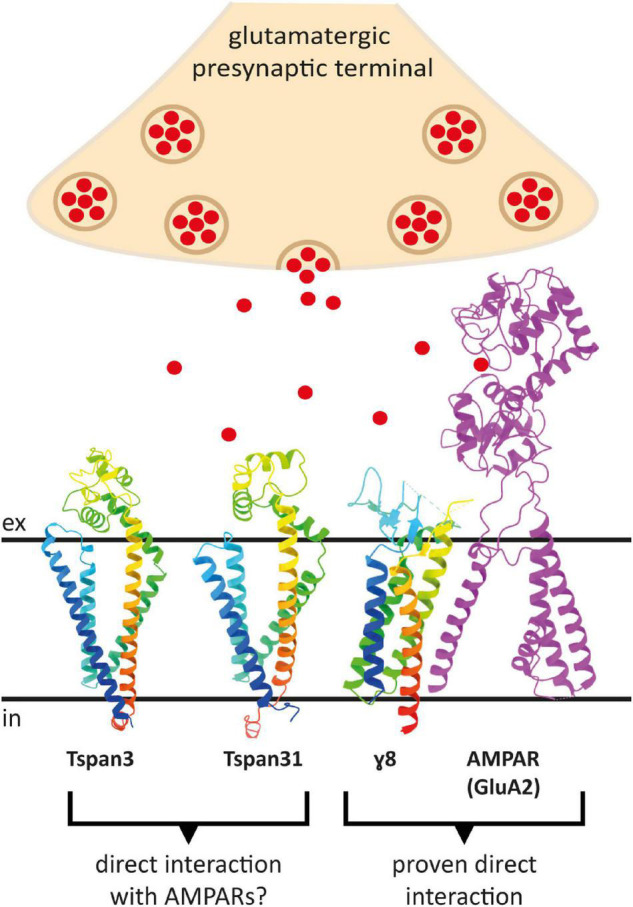
Putative direct interactions of tetraspanins with AMPARs at the postsynaptic membrane of a glutamatergic synapse. Shown is a GluA2 subunit in the D position in magenta, which has been proven to interact with the TARP γ8 (in rainbow colors) (right). To the left are the AlphaFold prediction models of Tspan3 and Tspan31, also colored in rainbow colors, which may also affect AMPAR function in a TARP-like manner. Red dots represent glutamate molecules. The proteins are displayed in ribbon style. The AMPAR subunit GluA2 lacks the C-terminus as well as the amino terminal domain (ATD) (PDB model: 7LEP) (Yu J. et al., 2021).

To predict which other tetraspanins might play an important role in the glutamatergic synapse, we superimposed structures of all other tetraspanins (predicted by AlphaFold) with Tspan7 using the ChimeraX program. Out of all aligned structures, four tetraspanins (Tspan3, Tspan6, Tspan13, and Tspan31) stand out (Figure 9) because of their small Root mean square distance (RMSD) value in comparison to other tetraspanins when aligned with Tspan7 (Table 2). On the basis of the small RMSD values which indicate high structural similarity of aligned proteins (Carugo, 2007), we hypothesize that these particular tetraspanins (Tspan3, Tspan13, and Tspan31) may share functional characteristics with Tspan7, that have already been demonstrated for Tspan6 (Salas et al., 2017) and thus may play an important role in the CNS. Both direct and indirect interactions with CNS proteins are conceivable. Figure 10 shows a hypothetical direct interaction of Tspan3 and Tspan31 with the AMPAR subunit GluA2 at a glutamatergic synapse. Next to it, TARP γ8 is shown, which functions in a similar manner as γ2 with respect to its AMPAR-modulating effects. Further investigation is required to characterize their functional properties in relevance to the CNS expression pattern of these tetraspanins.

**TABLE 2:** RMSD values (in Å) for human Tspan1-Tspan33 in alignment with Tspan7.

Tspan7 aligned with	RMSD between superimposed atoms (angstroms)	Tspan7 aligned with	RMSD between superimposed atoms (angstroms)
**Tspan1**	0.983	**Tspan18**	1.008
**Tspan2**	1.019	**Tspan19**	1.237
**Tspan3**	**0.90**	**Tspan20**	1.161
**Tspan4**	1.029	**Tspan21**	1.089
**Tspan5**	1.077	**Tspan22**	1.038
**Tspan6**	**0.774**	**Tspan23**	1.113
**Tspan7**	**0.000**	**Tspan24**	1.005
**Tspan8**	1.145	**Tspan25**	1.144
**Tspan9**	1.158	**Tspan26**	1.227
**Tspan10**	1.159	**Tspan27**	1.459
**Tspan11**	1.092	**Tspan28**	1.050
**Tspan12**	1.041	**Tspan29**	1.062
**Tspan13**	**0.829**	**Tspan30**	1.042
**Tspan14**	1.080	**Tspan31**	**0.854**
**Tspan15**	0.960	**Tspan32**	1.028
**Tspan16**	1.155	**Tspan33**	1.136
**Tspan17**	1.024		

*All tetraspanin structures were obtained from AlphaFold and aligned using Chimera-X. Smaller RMSD values indicate a higher similarity between the aligned protein structures. The color scheme used to highlight the values in the table refers to Figure 9, so Tspan7, which served as the template for the overlay, is shown in blue. The tetraspanins Tspan3, Tspan6, Tspan13, and Tspan31, closely related to Tspan7 according to the calculated RMSD values, are highlighted in red with bold RMSD numbers. An overlay of Tspan6 and Tspan7 is shown in Figure 7.*

## Conclusion and Perspectives

Studies in the past two decades have deepened our knowledge about the role of tetraspanins in diverse cellular processes. Although there has been considerable progress in understanding the relation between tetraspanins and its partner molecules involved in the regulation of numerous cellular functions, not much is known about structure-related functions, interaction, expression, and localization of tetraspanins in association with glutamate receptors.

Data from “The Human Protein Atlas” database indicated that Tspan3, Tspan5, Tspan6, Tspan7, Tspan15, Tspan28, Tspan29, and Tspan30 RNA is detected in functionally relevant quantities in different brain regions. According to the literature, all these tetraspanins have significant role in CNS, whereas only for Tspan6 and Tspan7 it has been proven to interact with glutamate receptors. However, since only three tetraspanin structures have been resolved so far, therefore we suggest that the structural similarity of predicted tetraspanin structures with the Tspan7 might indicate other unexplored members of tetraspanin family with the potential to contribute to the organization of the glutamatergic synapse. Moreover, beside the reported role of Tspan6 and its closest paralog Tspan7 at AMPAR-mediated synaptic transmission, three additional tetraspanins, Tspan3, Tspan13, and Tspan31 also point toward their potential involvement in important processes at the glutamatergic synapse based on their smaller RMSD value in comparison to other tetraspanins.

In addition, Tspan7 mutation and Tspan6 mRNA upregulation has been linked to intellectual disability and Alzheimer’s disease, respectively, therefore, understanding the regulation of glutamatergic synapses by tetraspanins may help to develop new targets for therapeutic interventions of several CNS diseases. Considering the abundant expression and broad spectrum of functions of tetraspanins in CNS, further investigation is required into the synaptic role of structurally related members of the tetraspanin family as well as understanding the mechanism of their actions as potential auxiliary subunits of AMPARs.

## Author Contributions

All authors contributed to the article and approved the submitted version.

## Conflict of Interest

The authors declare that the research was conducted in the absence of any commercial or financial relationships that could be construed as a potential conflict of interest.

## Publisher’s Note

All claims expressed in this article are solely those of the authors and do not necessarily represent those of their affiliated organizations, or those of the publisher, the editors and the reviewers. Any product that may be evaluated in this article, or claim that may be made by its manufacturer, is not guaranteed or endorsed by the publisher.
